# LPS-Induced Delayed Preconditioning Is Mediated by Hsp90 and Involves the Heat Shock Response in Mouse Kidney

**DOI:** 10.1371/journal.pone.0092004

**Published:** 2014-03-19

**Authors:** Tamás Kaucsár, Csaba Bodor, Mária Godó, Csaba Szalay, Csaba Révész, Zalán Németh, Miklós Mózes, Gábor Szénási, László Rosivall, Csaba Sőti, Péter Hamar

**Affiliations:** 1 Institute of Pathophysiology, Semmelweis University, Budapest, Hungary; 2 Department of Medical Chemistry, Semmelweis University, Budapest, Hungary; 3 Hungarian Academy of Sciences-Semmelweis University, Nephrology Research Group, Budapest, Hungary; Fred Hutchinson Cancer Research Center, United States of America

## Abstract

**Introduction:**

We and others demonstrated previously that preconditioning with endotoxin (LPS) protected from a subsequent lethal LPS challenge or from renal ischemia-reperfusion injury (IRI). LPS is effective in evoking the heat shock response, an ancient and essential cellular defense mechanism, which plays a role in resistance to, and recovery from diseases. Here, by using the pharmacological Hsp90 inhibitor novobiocin (NB), we investigated the role of Hsp90 and the heat shock response in LPS-induced delayed renal preconditioning.

**Methods:**

Male C57BL/6 mice were treated with preconditioning (P: 2 mg/kg, ip.) and subsequent lethal (L: 10 mg/kg, ip.) doses of LPS alone or in combination with NB (100 mg/kg, ip.). Controls received saline (C) or NB.

**Results:**

Preconditioning LPS conferred protection from a subsequent lethal LPS treatment. Importantly, the protective effect of LPS preconditioning was completely abolished by a concomitant treatment with NB. LPS induced a marked heat shock protein increase as demonstrated by Western blots of Hsp70 and Hsp90. NB alone also stimulated Hsp70 and Hsp90 mRNA but not protein expression. However, Hsp70 and Hsp90 protein induction in LPS-treated mice was abolished by a concomitant NB treatment, demonstrating a NB-induced impairment of the heat shock response to LPS preconditioning.

**Conclusion:**

LPS-induced heat shock protein induction and tolerance to a subsequent lethal LPS treatment was prevented by the Hsp90 inhibitor, novobiocin. Our findings demonstrate a critical role of Hsp90 in LPS signaling, and a potential involvement of the heat shock response in LPS-induced preconditioning.

## Introduction

Acute kidney injury (AKI) is a leading cause of death in the intensive care units. The incidence of AKI may exceed that of stroke [1] and even if patients survive there is a high risk of developing or exacerbating chronic kidney disease (CKD) [1], [2]. The main cause of AKI is ischemia-reperfusion injury (IRI)-induced acute tubular necrosis (ATN) [3], [4]. The kidney is an important shock organ, often being responsible for the death of septic patients. Sepsis is the most common cause of AKI in critically ill patients [5]. In septic patients bacterial lipopolysaccharide (endotoxin, LPS) can significantly contribute to AKI [6], [7].

Currently there are no effective therapeutic strategies to reduce AKI [8]. Delayed preconditioning is an attractive method to reduce IRI of different organs, such as the heart, brain or kidney. A better understanding of the molecular mechanisms behind delayed preconditioning could enable the development of new therapeutic strategies to pharmacologically induce tissue resistance to AKI.

Delayed preconditioning elicited by a stressful stimulus promotes survival by increasing tolerance to a subsequent lethal stress. Preconditioning LPS treatment prevents death from a subsequent lethal LPS dose [9]. Moreover, the endotoxin-induced cross-tolerance to IRI has been described in the heart [10] and brain [11]. Based on this principle, we have previously established a model for delayed-type LPS cross-tolerance to renal IRI. We demonstrated that LPS was capable of inducing delayed preconditioning, and protected from a subsequent lethal LPS dose or lethal renal IRI [12]. Mechanisms of this delayed cross-tolerance have been investigated since then [13], [14]. Molecular interactions between renal IRI and LPS have been also demonstrated at the mRNA level [15]; however, the exact mechanism of LPS-induced delayed preconditioning remains unclear.

The heat shock response is an ancient and essential cellular survival mechanism that operates via a heat shock transcription factor 1 (HSF1)-dependent induction of heat shock proteins (HSPs). The major heat shock proteins, such as Hsp70 and Hsp90 confer tolerance to a plethora of stresses by maintaining the conformational homeostasis and exerting anti-apoptotic effects [16], [17]. Besides, Hsp90 plays specific roles, such as keeping HSF1 in an inactive form, and chaperoning various signaling molecules. Along with Hsp70 Hsp90 mediates LPS-signaling as a part of the LPS receptor cluster [18]–[20]. The Hsp90 function involves ATP-binding and hydrolysis coordinated by the N- and C-terminal domains of the chaperone [21]. N-terminal Hsp90 inhibitors, exemplified by geldanamycin, are promising anti-cancer agents, currently in clinical trials [22]. They potently induce the heat shock response, which is a major drawback in antitumor therapy but are extensively utilized in a number of degenerative diseases such as ischemic diseases and organ transplantation [17], [23]. It has also been demonstrated that geldanamycin protects renal cells from oxidative stress and reduce renal ischemia-reperfusion injury [24], [25]. In contrast, the coumarin antibiotic novobiocin (NB), a C-terminal Hsp90 antagonist inhibits Hsp90 function without induction of the heat shock response [22], [26], [27]. Utilizing these excellent properties of NB, we applied NB treatment to study the involvement of Hsp90 and the heat shock response in LPS-induced delayed preconditioning in mice.

Our results demonstrate that the Hsp90 inhibitor NB completely prevented LPS-induced delayed preconditioning to lethal LPS administration in mice. Besides, NB prevented the induction of Hsp70 and Hsp90 protein induction in response to LPS. Thus, our data support a critical role of Hsp90 and the heat shock response in endotoxin-tolerance, but also warn that Hsp90 inhibition with novobiocin may be deleterious in sepsis.

## Methods

### Ethics Statement

By survival studies humane endpoints were used to minimize suffering. Animals were observed and weighed after potentially lethal interventions including LPS regimen every morning. In case clinical signs of distress were recognized, the animals were euthanized by cervical dislocation performed by the PI trained in this technique. Clinical signs of endotoxin shock included reduced locomotion, signs of diarrhea, piloerection and a body weight loss exceeding 40% of the initial body-weight or a reduction below 18 g. All intraperitoneal injections and sacrifice for organ removal were performed under ether anesthesia. The protocol was reviewed and approved by the Governmental Animal Care Agency and the Research Committee Board of Semmelweis University 53/2001. (V.31.) ET.).

### Animals

Male C57BL/6 mice (Charles River, Germany) weighing 27.6±1.8 g were used. All animals were housed under standard specified pathogen-free conditions (light on 08:00–20:00 h; ambient relative humidity: 40–70%, 22±1°C), and had free access to tap water and chow (Altromin standard diet, Germany). After 1 week of acclimatization mice were randomized into eight experimental groups with similar average body weight at inclusion.

### Treatments

Mice were treated intraperitoneally (ip.) under short ether (diethyl-ether, Reanal, Budapest, Hungary) anesthesia. Novobiocin (Promega, WI, USA) dose was 100 mg/kg. The preconditioning dose of LPS (Escherichia coli, Serotype 0111:B4; Sigma-Aldrich, Budapest, Hungary) was 2 mg/kg and the lethal dose of LPS was 10 mg/kg (ip.) as previously established by us [12]. The animals were treated on 2 consecutive days (Table 1). Survival was observed in five animals in each treatment group. Another five animals in each group were sacrificed for the determination of renal HSP expression.

**Table 1:** Treatment protocols for LPS preconditioning.

Group	Day 1	Day 2
**Saline**	0.1 ml/10 g saline	0.1 ml/10 g saline
**NB**	0.1 ml/10 g saline	100 mg/kg NB
**LPS(p)**	2 mg/kg LPS	0.1 ml/10 g saline
**LPS(p+L)**	2 mg/kg LPS	10 mg/kg LPS
**LPS(p)+ NB**	2 mg/kg LPS	100 mg/kg NB
**LPS(L)**	0.1 ml/10 g saline	10 mg/kg LPS
**NB+ LPS(L)**	100 mg/kg NB	10 mg/kg LPS
**LPS/NB+ LPS(L)**	2 mg/kg LPS +100 mg/kg NB	10 mg/kg LPS

Saline: physiological saline, LPS: bacterial lipopolysaccharide endotoxin, p: preconditioning dose, L: lethal dose, NB: novobiocin.

### Detection of renal damage by plasma urea concentration

Renal function was evaluated by determination of urea retention at the time of organ harvesting. Blood urea levels were measured from 32 μl of whole blood with Reflotron® Urea test stripes (Roche Diagnostics GmbH, Mannheim, Germany) on the Reflotron® Plus device (Roche) following the manufacturer's protocol.

### Organ harvest

Animals were sacrificed 24 hours after the second treatment. Harvest began with ether anesthesia and injection of heparin (500 U/animal ip., Heparin Biochemie GmbH, Schaftenau, Austria) of mice. Blood was removed from parenchymal organs by injection of 20 ml ice cold Hank's Buffered Salt Solution (Sigma-Aldrich, Budapest, Hungary) through the left ventricle using a 20 ml syringe and an 18 G needle. Following perfusion, kidneys were removed and the upper pole of each kidney was fixed in 4% buffered formalin for one day, and then were dehydrated and embedded in paraffin (FFPE) for morphological analysis. The lower pole was snap frozen in liquid nitrogen and kept at −80°C for molecular investigations.

### Histological analysis

Four μm thick FFPE sections were deparaffinized, rehydrated and consecutively stained with haematoxylin and eosin (H&E) and periodic acid Shiff (PAS). Sections were examined in a blinded fashion. All photomicrographs were taken with Leica DC500 microscope.

The extent of ischemic renal damage was estimated using a previously published scoring system [12]. Severity of tubular injury was determined by changes of nucleus morphology, tubular cell vacuolization, detachment or hyalinization. Periodic acid Schiff (PAS) stained kidney samples were scored under x200 absolute magnification, and a score from 0 to 3 was given for each tubular profile per field of view: 0 =  normal histology; 1 =  tubular cell swelling, mild-moderate brush border damage, vacuolization of tubular epithelial cells; 2 =  moderately dilated tubules, more severe brush border loss, edematous tubular epithelial cells, focally weak/lost nuclear staining; 3 =  total tubular necrosis, neutrophil granulocyte infiltration, no nuclear staining, dilated tubules with or without tubular cast formation in the lumen. Mean values were calculated from 10 fields of view.

### Kidney Hsp70, Hsp90α, Hsp90β and α-actin protein and mRNA expression

Kidney samples were snap-frozen in liquid nitrogen and stored at −80°C until use. Protein and mRNA of Hsp70 and Hsp90α and Hsp90β were determined by Western and Northern blotting, respectively.

### RNA extraction and Northern Blot Analysis

Samples were homogenized in TRIzol (Life Technologies, Gaithersburg, MD, USA), extracted with phenol-chloroform, and the RNA was precipitated with isopropanol. Total RNA (12 μg) was electrophoresed through a 1% formaldehyde-agarose gel and transferred to a Nytran membrane (Schleicher & Schuell, Keene, NH, USA). The membrane was ultraviolet cross-linked, baked at 80°C for 2 h, and hybridized for 1 h with Hybrisol I (Oncor, Gaithersburg, MD, USA). The following cDNAs were used: mouse Hsp70 detecting both Hsp72 and Hsc70 (Hsp73), (Kerstin Bellmann, Heinrich-Heine University, Düsseldorf, Germany), Hsp90α (kind gift of Yoshihiko Miyata, Kyoto University, Japan) and Hsp90β (kind gift of Attila Sebe, Semmelweis University, Institute of Pathophysiology, Hungary), and human S14 ribosomal protein (plasmid no. 59247, American Type Culture Collection, Manassas, VA, USA) as a housekeeping gene. The Hsp70 and Hsp90 plasmids were PCR amplified (ICycler, Bio-Rad Hercules, CA, USA), run on an agarose gel to excise the amplicon. DNA was isolated (GeneElute Kit, Sigma-Aldrich, Budapest, Hungary) and inserted into a plasmid with InsTAclone PCR Product Cloning Kit (Fermentas, St. Leon-Rot, Germany). All plasmids were amplified in E. Coli and were isolated with the cesium chloride density gradient ultracentrifugation method [28], and the cDNA was excised. The cDNA inserts were labeled with 32P-dCTP (Amersham, Arlington Heights, IL, USA), using the Prime It II random prime labeling kit (Stratagene, La Jolla, CA, USA). The membranes were hybridized overnight at 42°C, and washed with PES buffer (40 mM sodium phosphate, pH 7.4, 1 mM ethylene-diamine-tetra-acetic acid, 0.5% sodium dodecyl sulfate) twice at room temperature for 15 min and once at 55°C for 15 min. The radioactive signal was detected and quantified using a Phosphor Imager Molecular Imager FX (Bio-Rad, Hercules, CA, USA).

### Protein preparation and Western blotting

For Western blotting analysis the excised specimens were immediately dropped into liquid nitrogen. Western blotting was performed as described previously [29]. Briefly, frozen tissue samples were homogenized in ice-cold lysis buffer in 15 ml centrifuge tubes by a mechanical homogenizer. The lysis buffer contained 25 mM Tris (pH 7,4), 1% NP-40, 100 mM NaCl, 4 mM EDTA, 1 mM NaVO_4_, 10 mM NaF, 1 mM DTT (all chemicals were purchased from Sigma-Aldrich, Budapest, Hungary) and supplemented with 50x protease inhibitor cocktail (BD Biosciences Pharmingen, San Diego, CA, USA). Protein concentrations were determined by the Bradford method. Tissue lysates were mixed 1∶1 with 2x Laemmli sample buffer and boiled for 5 min at 95°C. Equal amounts of protein for each sample were loaded onto 9% polyacrylamide gels (Bio-Rad, Hercules, CA, USA), separated by SDS-PAGE, and transferred to nitrocellulose membranes (Bio-Rad, Hercules, CA, USA). The membranes were blocked with 5% non-fat dry milk and incubated with a monoclonal anti-Hsp72 (Stressgen Biotechnologies, Victoria, Canada), polyclonal anti-Hsp90 (recognizing both the alpha and beta isoforms (kind gift of Yoshihiko Miyata, Kyoto University, Kyoto, Japan) and α-aktin (Sigma-Aldrich, Budapest, Hungary) antibodies and HRP-conjugated secondary antibodies. The membranes were developed with an enhanced chemiluminescence detection system (PerkinElmer, Wellesley, MA, USA) and analyzed by densitometry.

### Statistical analysis

All continuous data are expressed as mean+SD unless otherwise stated. Comparisons among groups used one-way analysis of variance (ANOVA) followed by Tukey's multiple comparisons test for between-groups comparisons. The Kruskal–Wallis one-way analysis of variance by ranks was performed if Bartlett's test indicated heterogeneity of variances, and comparisons between groups used Dunn's test. Nonparametric data were tested using Mann-Whitney analysis of ranks. Survival curves were compared by Kaplan- Meyer log-rank test. All tests were conducted at the two-sided 5% significance level. Statistical analysis was performed using GraphPad Prism5 (GraphPad Software Inc., San Diego, CA, USA).

## Results

### Novobiocin reversed the preconditioning-induced survival from lethal LPS shock

**Figure 1:**
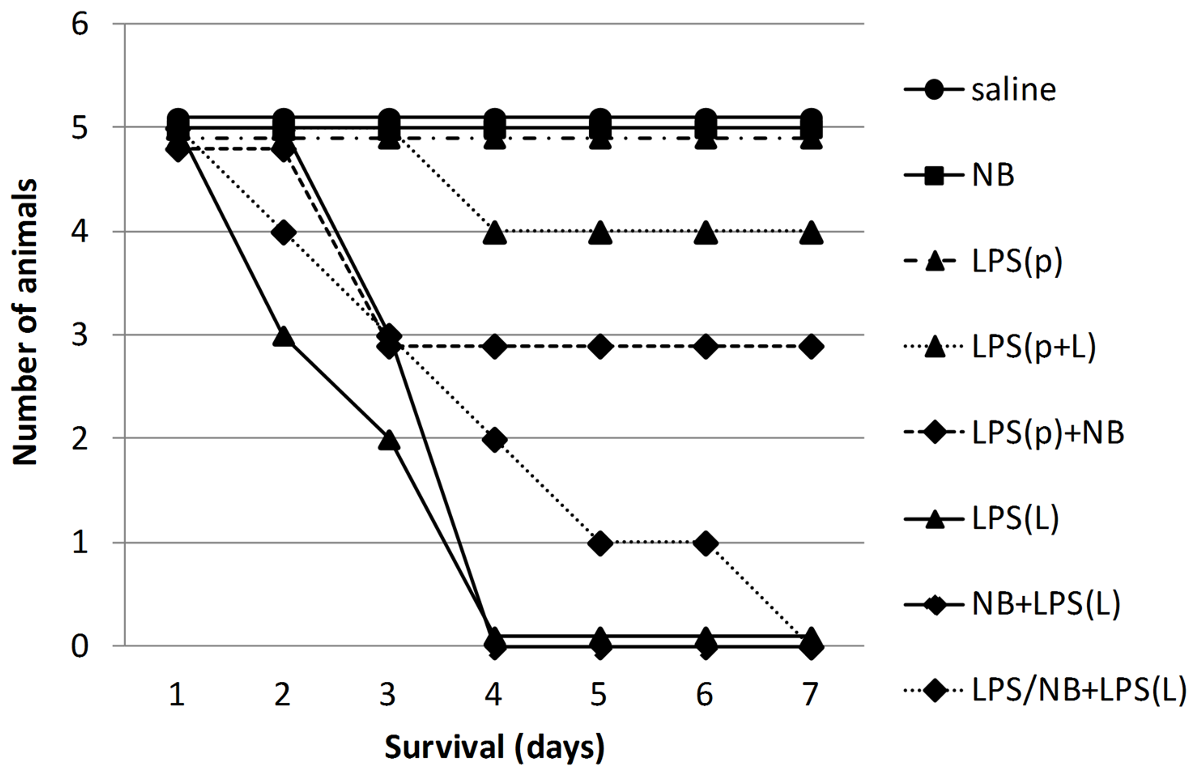
Survival. The control group treated with saline (saline) is represented with filled circles and solid line, the group treated with novobiocin (NB) is represented with filled squares and solid line, the groups treated with LPS are represented with triangles and dashed line (LPS(p)), solid line (LPS(L)) and dotted line (LPS(p+L)), the groups treated with LPS + NB are represented with diamonds and solid line (NB + LPS(L)) and dotted line (LPS/ND + LPS(L)).

First, we investigated how inhibition of Hsp90 with novobiocin (NB) influenced LPS-induced preconditioning in male mice (Figure 1). All mice treated with saline, or NB without LPS or a preconditioning dose of LPS (LPS(p)) survived. However, all mice died within one week after treatment with a lethal dose of LPS (LPS(L)) (p<0.01 vs. saline). Preconditioning with LPS prevented death from a subsequent lethal LPS dose as only one animal died in this group (LPS(p+L)) (p<0.01 vs. LPS(L)). However, pretreatment with NB (LPS/NB+LPS(L)) prevented the preconditioning effect of LPS(p) as all mice died in this group (p<0,01 vs. LPS(p)). Thus, NB prevented delayed preconditioning (Figure 1). Furthermore, NB sensitized to LPS to some extent as two animals also died after treatment with LPS(p) and NB (LPS(p)+NB) (ns).

**Figure 2:**
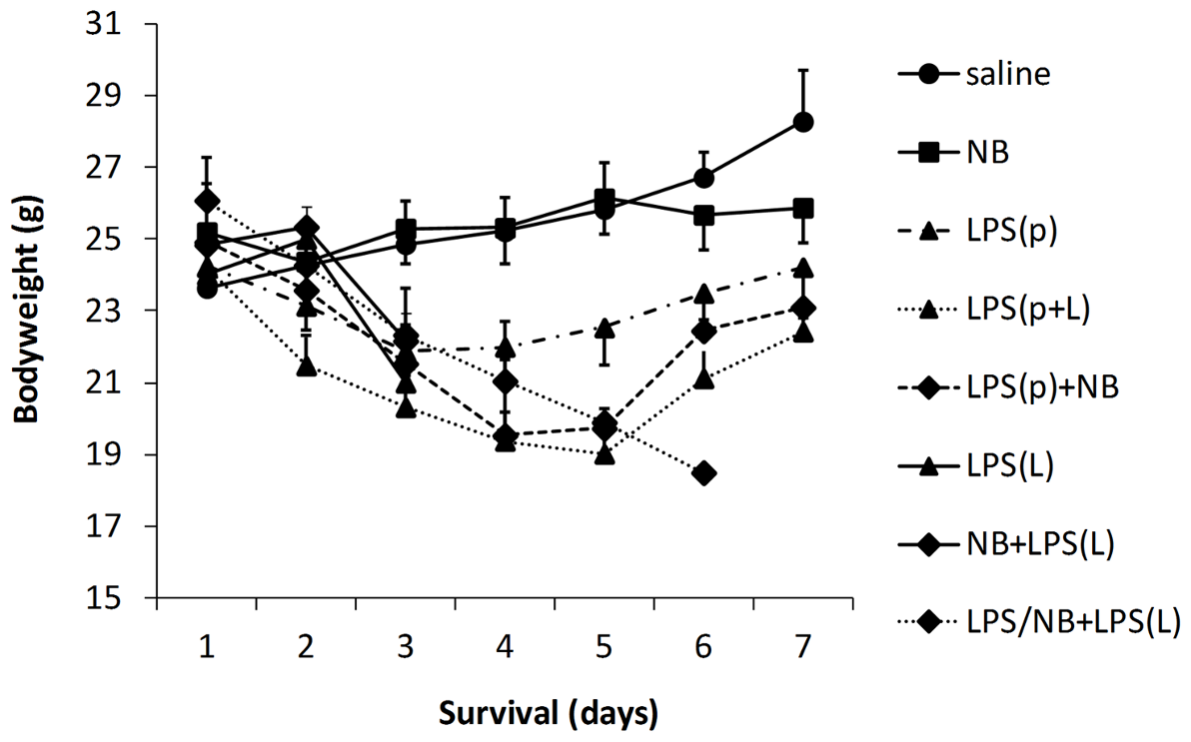
Bodyweight. The control group treated with saline (saline) is represented with filled circles and solid line, the group treated with novobiocin (NB) is represented with filled squares and solid line, the groups treated with LPS are represented with triangles and dashed line (LPS(p)), solid line (LPS(L)) and dotted line (LPS(p+L)), the groups treated with LPS + NB are represented with diamonds and solid line (NB + LPS(L)) and dotted line (LPS/ND + LPS(L)).

The saline-treated control animals (saline) demonstrated a steady rate of bodyweight gain (Figure 2). NB alone did not significantly influence weight gain. Treatment with endotoxin at a preconditioning dose (LPS(p)) induced about 20% bodyweight loss within the first 3 days in all animals compared to animals not receiving LPS. However, after 3–4 days, the animals started to recover from the preconditioning dose of endotoxin (LPS(p)). All animals treated with a lethal dose of LPS (LPS(L)) lost weight at a faster rate than those mice treated with LPS(p) and died (Figure 2). Treatment with a low dose of LPS induced delayed preconditioning as animals treated with a subsequent lethal dose of LPS (LPS(p+L)) initially lost weight but recovered from day 5 after treatment. If the lethal LPS dose was preceded with NB, all animals lost weight and died within 3 days (NB+LPS(L)). Also, NB administered simultaneously with the preconditioning dose of LPS prevented delayed preconditioning as animals did not recover from a subsequent treatment with LPS(L), but lost weight and died (LPS/NB+LPS(L)) (Figure 2).

### Novobiocin prevented preconditioning-induced protection of renal function and histology

**Figure 3:**
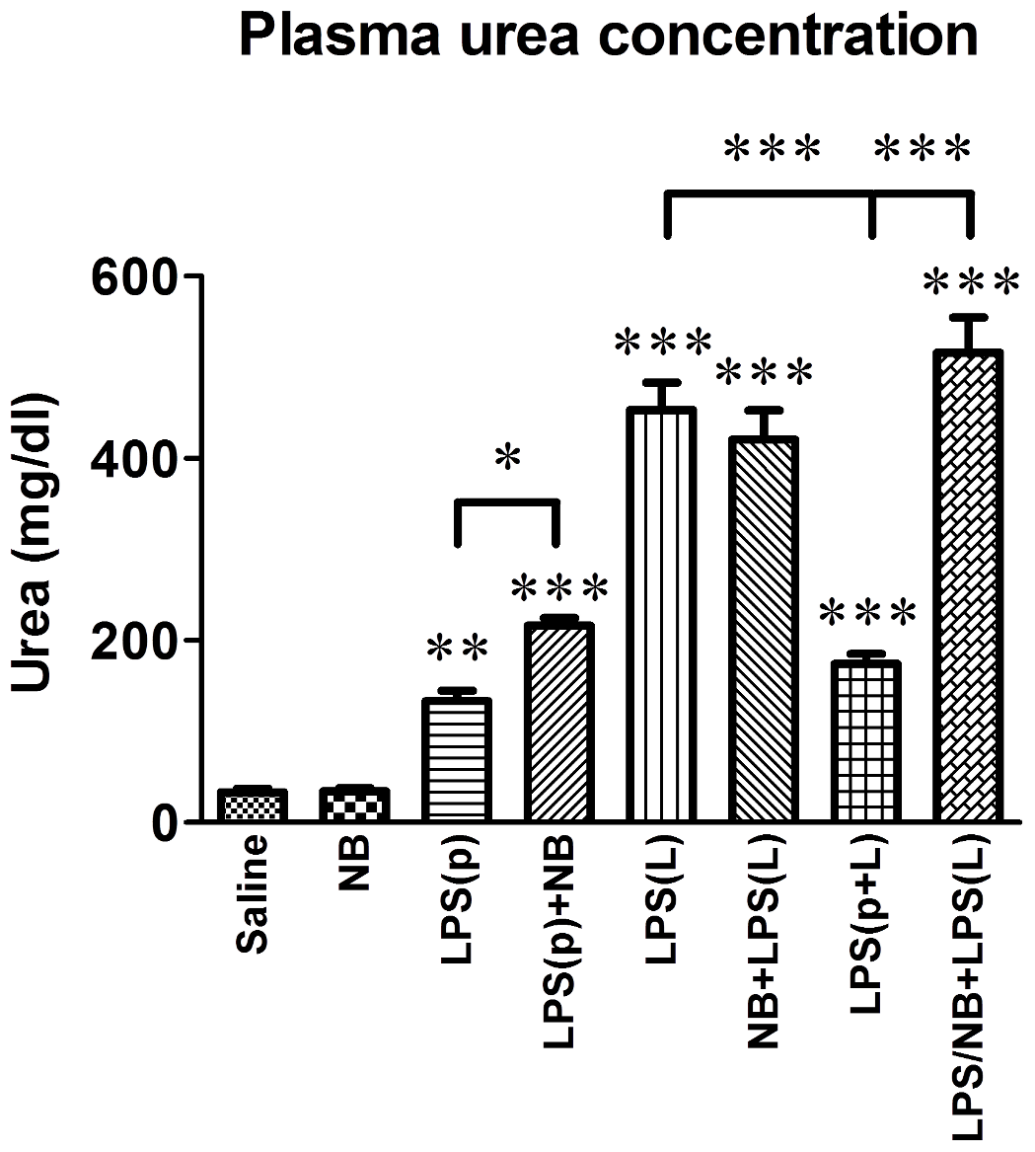
Plasma urea levels at the time of organ harvest i.e. at 24 hours after the second treatment. LPS: bacterial lipopolysaccharide endotoxin, p: preconditioning, L: lethal, NB: novobiocin. *, **, ***: p<0.05, 0.01, 0.001 vs. the saline-treated controls, respectively, or between the groups indicated.

As a sign of endotoxin shock-induced renal failure serum urea was elevated at 24 hours after the second injection (Figure 3). Compared to the saline-treated control mice, NB alone did not increase serum urea, i.e. it did not impair renal function. The preconditioning dose of LPS (LPS(p)) induced a temporary but significant elevation in serum urea. Preconditioning was obvious in mice treated with a preconditioning and a subsequent lethal LPS dose (LPS(p+L), as the preconditioning dose of LPS prevented the increase in serum urea that was caused by the lethal LPS dose. NB prevented preconditioning: a concomitant NB administration with LPS(p) (LPS/NB+LPS(L)) resulted in a similar increase of serum urea to that caused by the lethal LPS dose (Figure 3).

**Figure 4:**
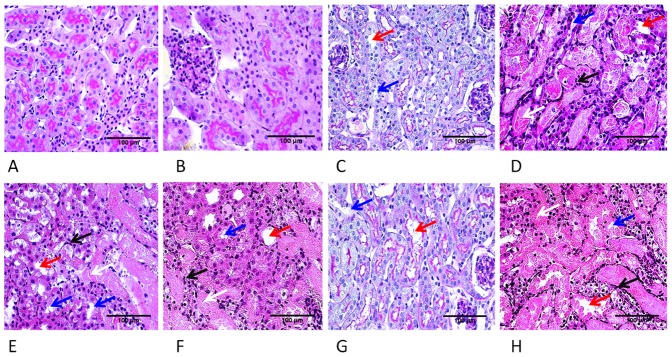
Representative photomicrographs and tubular damage index 24 hours after the second treatment. Photomicrographs: A) Saline B) Novobiocin C) LPS(p), D) NB+LPS(L), E) LPS(L), F) LPS(p)+NB, G) LPS(p+L), H) LPS/NB+LPS(L). J) Tubular damage score (PAS, 400x). Arrows indicate: dilatation of proximal tubules (red arrow), brush border loss (blue arrow), tubular casts (white arrow), neutrophil infiltration (black arrow). LPS: bacterial lipopolysaccharide endotoxin, p: preconditioning, l: lethal, NB: novobiocin. *, **, ***: p<0.05, 0.01, 0.001 vs. the saline-treated controls, respectively, or between the groups indicated.

Renal histology was normal in mice treated with saline or NB (Figure 4) as high cylindrical tubular epithelial cells and intact brush border was observed on PAS-stained slides. Various degree of tubular damage was obvious in all LPS-treated mice; however, the histological damage index was low in mice treated with only a preconditioning (LPS(p)) or a preconditioning and a subsequent lethal dose of LPS (LPS(p+L). Preconditioning with a low dose of LPS preserved renal morphology but the protective effect of preconditioning was diminished by a simultaneous treatment with NB (Figure 4).

### Novobiocin inhibited LPS-induced Hsp70 and Hsp90 protein expression

**Figure 5:**
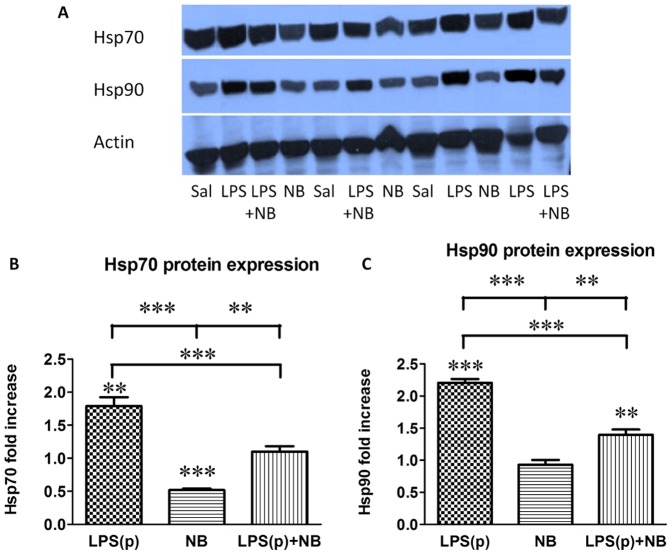
Western blot analysis of Hsp70 (A), and Hsp90 (B) protein expression. Results are normalized to actin protein expression, and presented as fold increase relative to the saline-treated group with its mean set to 1. **, ***: p<0.01, 0.001 vs. the saline-treated controls, respectively, or between the groups indicated (n = 6/group).

In order to explore the molecular background of LPS-induced preconditioning and how NB prevented preconditioning, we investigated the effects of NB on heat shock protein expression with Western blot analysis. The preconditioning dose of LPS significantly stimulated (doubled) Hsp70 and Hsp90 (Figure 5) expression compared to the saline-treated mice. NB administered alone significantly reduced Hsp70 but not Hsp90 protein expression vs. saline-treated mice. LPS(p)-induced stimulation of Hsp70 and Hsp90 was inhibited by simultaneous administration of NB.

### Novobiocin increased Hsp90 mRNA expression and prevented LPS-induced Hsp70 mRNA expression

**Figure 6:**
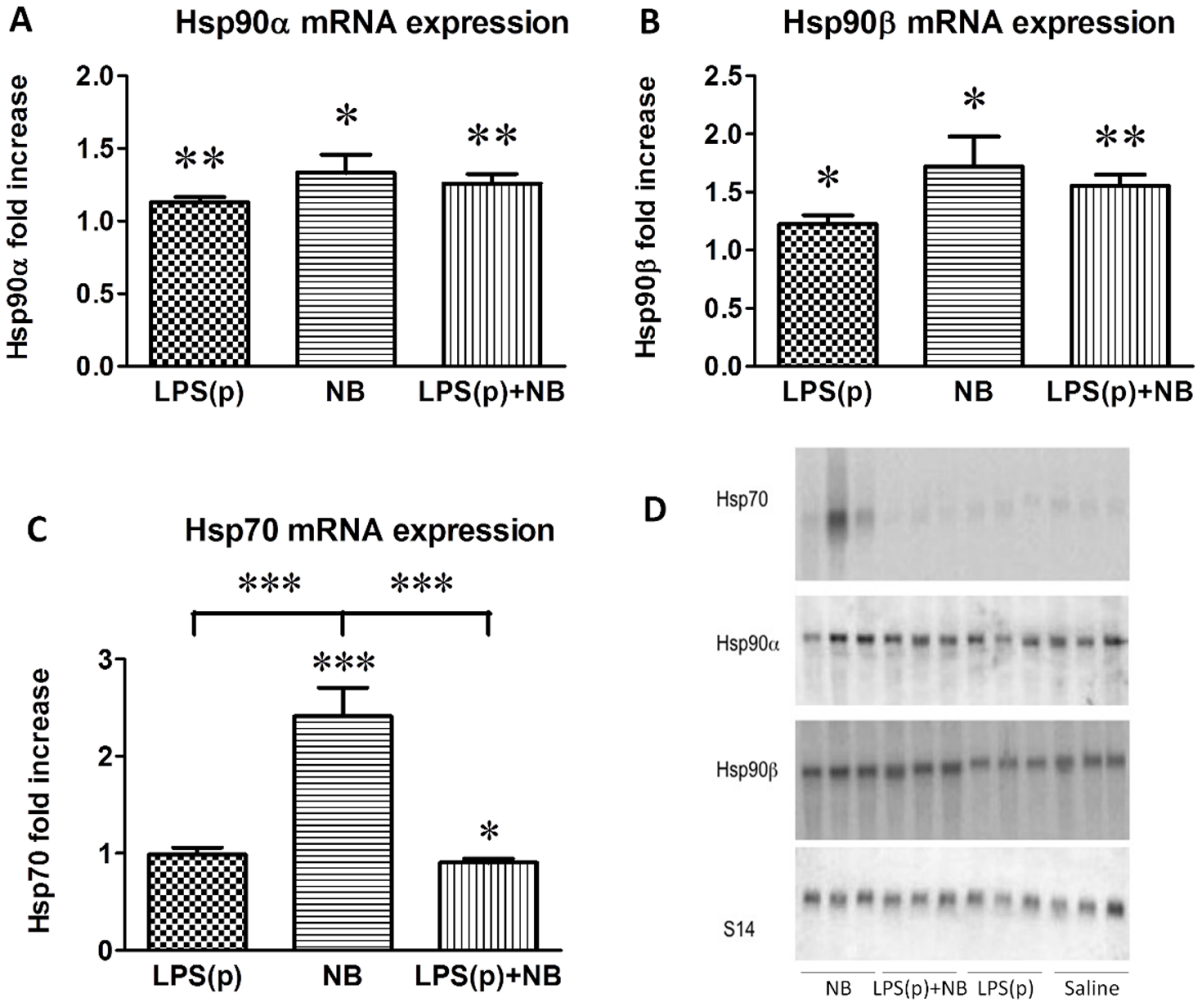
Northern blot analysis of Hsp70 (A), Hsp90α (B) and Hsp90β (C) mRNA expression. Results are normalized to S14 expression and presented as fold increase relative to the saline-treated group with its mean set to 1. *, **, ***: p<0.05, 0.01, 0.001 vs. the saline-treated controls, respectively, or between the groups indicated (n = 6/group).

To explore the mechanism of LPS-induced increase in HSP protein expression, we measured Hsp70, Hsp90α and Hsp90β mRNA levels by Northern blotting. LPS(p) significantly stimulated Hsp90α and Hsp90β but not Hsp70 mRNA levels (Figure 6), probably due to the HSF1-mediated transcriptional induction of the heat shock response. Interestingly, NB also evoked a significant induction of all HSP mRNAs. The stimulating effect of NB was strongest in the case of Hsp70 mRNA and was similar to that of LPS(p) in the cases of Hsp90α and Hsp90β. LPS(p)-evoked heat shock response was not inhibited by NB at the mRNA level of Hsp90α and Hsp90β. Neither were the Hsp90 stimulatory effects of LPS and NB additive (Figure 6). In summary, NB treatment stimulated Hsp70 and Hsp90 mRNA. Taken together with the protein level studies, these results suggest that in the background of diminished preconditioning LPS-induced Hsp70 and Hsp90 protein expression was inhibited by NB at the posttranscriptional level.

## Discussion

In our previous study, repeated pretreatment with LPS protected mice from a lethal renal ischemia/reperfusion injury [12]. In the present study we demonstrated that treatment with a preconditioning dose of LPS conferred protection from kidney damage and consequent death caused by subsequent treatment with a lethal dose of LPS. Further, we demonstrated that delayed preconditioning with LPS was abrogated by concomitant treatment with the Hsp90 inhibitor NB. This study provides evidence that delayed preconditioning is accompanied by the induction of heat shock proteins Hsp70 and Hsp90, and inhibition of Hsp90 function by NB prevents delayed preconditioning of the kidney. Similarly to previous studies, we confirmed that LPS induced acute renal failure [30], and this acute renal failure was the main cause of death in mice treated with LPS.

Hsp70 was the first discovered heat shock protein [31] and is the most ubiquitous and highly conserved HSP [32]. Heat shock protein family members assist protein folding and protect unfolded chains from nonspecific interactions such as aggregation, and proteins from damage and denaturation during cell stress such as dehydration, temperature changes or hypoxia [33]. However, HSPs are also necessary for the ubiquitination and breakdown of damaged or misfolded proteins by the proteasome [34], [35]. Thus, HSPs are involved in both the creation, protection, maintenance of protein homeostasis, but also the destruction of damaged proteins. Hsp90 is a hub in the chaperone network maintaining healthy protein turnover in cells upon insults such as endotoxin shock [36].

Both Hsp70 and Hsp90 are present in the kidney [37] and thought to play a central role in preconditioning [38]. The present study corroborates these previous observations, as a preconditioning LPS dose induced Hsp70 and Hsp90 protein and Hsp90 mRNA expression in the renal tissue that later protected from an otherwise lethal endotoxemia. A possible explanation for the lack of Hsp70 mRNA upregulation, despite doubling of the Hsp70 protein level 24 hours after LPS treatment could be, that the mRNA peak is earlier than investigated in the present study. In previous studies, LPS-induced upregulation of Hsp70 and Hsp90 has been demonstrated in the blood of septic patients [39], or in different organs such as the lung [40]–[42], skin [43] or macrophages [44] of LPS-treated rodents. Similarly to our present study, heat shock proteins were upregulated in the kidney upon LPS treatment [38]. Furthermore, Hsp70 and Hsp90 protein upregulation had a central protective role in preconditioning against endotoxin shock [40], [45]. Similarly to our results, local upregulation of Hsp70 in the kidney and other organs during preconditioning had a key role in protecting the kidney, lung and liver from multi-organ failure [46]. On the other hand, exercise preconditioning protected the kidney from IRI even in Hsp70 KO mice [47], and pretreatment with monophosphoryl lipid A, an endotoxin analogue, protected the heart from IRI in rabbits without an increase in cardiac Hsp70 protein expression [48]. These data taken together with ours indicate that Hsp90 may be more important mediator of preconditioning than Hsp70.

Furthermore, the involvement of Hsp mediated processes have been reported recently in the background of other popular preconditioning theories such as nitric oxide (NO) [49] or regulatory T-cell mediated protection [50].

NB a well-known inhibitor of Hsp90 function inhibited LPS induced Hsp70, and Hsp90 protein expression. These findings corroborate previous reports and demonstrate a critical role of Hsp90 in LPS signaling [18].

Moreover, NB antagonized the preconditioning-induced survival, amelioration of kidney morphology and function as well as the stimulatory effects of LPS on Hsp70 and Hsp90 protein expression. This inhibition of LPS-induced Hsp70 and Hsp90 protein upregulation may have been responsible for the prevention of delayed preconditioning and death of mice after pretreatment with NB. An intriguing finding is, that NB treatment alone significantly stimulated Hsp70 and Hsp90 mRNA expression but inhibited protein expression. NB a selective Hsp90 inhibitor might inhibit Hsp70 protein post-transcriptionally by micro RNAs, as it has been suggested that Hsp90 may be important in miRNA post-transcriptional gene-expression regulation [51].

A recent review suggests that pharmacologic Hsp90 inhibitors may protect the kidney from ischemia via the induction of the heat shock response [25]. These results raise the idea that the heat shock response is a mediator of preconditioning LPS treatment. Further studies are needed to address this hypothesis. Importantly, our study warns, that Hsp90 inhibitors, such as NB, that abrogate the heat shock response should be used with caution in septic patients with renal involvement.
